# A Comparison of Transfer Learning Metaphyseal Sign Diagnostic Models for Kashin-Beck Disease Based on X-rays of Children’s Hands

**DOI:** 10.7759/cureus.78235

**Published:** 2025-01-30

**Authors:** Ge Li, Hong Zhang, Ping Chen, Yunlong Song, Yuchen Zhang, Chao Li

**Affiliations:** 1 Disease Control and Prevention, Shaanxi Provincial Center for Disease Control and Prevention, Xi'an, CHN; 2 Occupational and Environmental Health, Xi'an Jiaotong University, Xi'an, CHN; 3 School of Humanities and Social Science, Xi'an Jiaotong University, Xi'an, CHN; 4 Epidemiology and Public Health, Shaanxi Provincial Tuberculosis Prevention and Control Hospital, Xi'an, CHN; 5 Orthopedics, Xijing Hospital, Xi'an, CHN

**Keywords:** deep learning, diagnostic models, kashin-beck disease, metaphyseal signs, transfer learning

## Abstract

Background

Kashin-Beck disease (KBD), prevalent in certain regions of the world, primarily affects children and is characterized by joint deformities. Timely screening and accurate diagnosis, heavily reliant on metaphyseal signs in X-rays, are crucial but challenging, especially in regions where specialist availability is scarce. Artificial intelligence (AI)-assisted diagnostic technology offers a valuable solution to streamline KBD screening, emphasizing its importance in enhancing diagnostic precision and efficiency.

Methods

This study developed and compared five deep learning models - KBV16, KBX, KBV19, KBIn, and KBM2 - to assist in diagnosing KBD by analyzing pediatric hand radiographs. We optimized these models with a dataset comprising 22,366 images, encompassing both metaphyseal positive and control groups. The models were trained and validated using Binary Cross-Entropy (BCE) and Accuracy (ACC) metrics.

Results

The KBV16 model outperformed the others, achieving an accuracy of 0.9563 on the validation set and 0.9535 on the test set. The implementation of data augmentation techniques, along with the meticulous selection of learning rates and batch sizes, significantly enhanced the models' performance.

Conclusion

This study presented a novel application of deep learning in KBD diagnosis, demonstrating the potential of AI models to enhance diagnostic precision. Notably, the KBV16 model emerged as a powerful tool for early detection of KBD. Future research should concentrate on refining these models for clinical use and integrating them into existing healthcare systems to improve medical services, particularly in medical resource-constrained regions.

## Introduction

Kashin-Beck disease (KBD) is an endemic, teratogenetic osteoarticular disease [1,2]. KBD exhibits a distinct geographic bias, and it is primarily found in specific regions of Asia, including southeastern Siberia, Russia, North Korea, and China [3]. In China, KBD is mainly distributed in a diagonal belt across the country, covering 13 provinces, including Heilongjiang, Shaanxi, and Tibet. This belt includes over 16,000 KBD-affected villages [4]. China is one of the countries with the most widespread and serious cases of KBD in the world [5]. The primary lesions of KBD are mainly symmetrical degeneration and necrosis of epiphyseal cartilage, epiphyseal plate cartilage, and articular cartilage during bone development, along with secondary degenerative osteoarthropathy [6]. The main clinical symptoms are joint pain, thickening, deformation, limited activity, muscle atrophy, and, in severe cases, short fingers, short limbs, and even dwarfism [7,8]. The diagnosis of KBD is mainly based on contact history, signs and symptoms, and hand radiographs. The alterations observed in the hand's metaphyseal region on X-ray imaging represent a distinctive diagnostic marker for this condition [9].

For decades, under the guidance of the Communist Party of China (CPC) and the Chinese government, a range of comprehensive preventative and control strategies have been implemented in light of various etiological theories. These measures, such as selenium supplementation, grain exchange, water exchange, and population relocation, have been adopted according to local conditions, and good effects have been achieved [10]. Thanks to these efforts, the epidemic of KBD in China began to decline in the early 1980s and reached basic control in 2009 [11], with only a small number of cases detected over the next 12 years [12,13].

As we examine the historical trajectory of KBD, there is a growing belief that the incidence of KBD will likely be predominantly sporadic in the future. However, the absence of a pandemic does not mean that new cases will not occur by chance. Insufficient clinical diagnostic experience with KBD could potentially impair the early detection of isolated cases, thereby impeding the disease's prevention and control efforts. Notably, the presence of multiple symmetrical depressions, deformities, and other alterations in the metaphyseal calcification zone, observed in hand X-ray films, is among the key diagnostic indicators for identifying KBD [9]. However, there is no model using artificial intelligence (AI) technology to assist in the diagnosis of X-ray metaphyseal signs of KBD.

Early diagnosis of KBD is crucial for preventing joint deformities and improving prognosis. However, traditional diagnostic methods rely on identifying metaphyseal signs in X-ray images, which require specialized radiological knowledge and experience. In areas with limited resources, the lack of specialized doctors poses a challenge for timely and accurate diagnosis. AI-assisted diagnostic technology can quickly analyze large amounts of imaging data and provide highly accurate diagnostic results, thus compensating for the shortage of specialized doctors. AI models can process large volumes of imaging data in a short time, reducing the workload of manual screening. For example, some deep learning-based algorithms can automatically identify KBD features in hand X-ray images, helping governments reduce the input of human resources. In some remote areas, medical resources are scarce, and AI diagnostic systems can serve as an effective auxiliary tool to help local doctors diagnose KBD more accurately, thereby optimizing the allocation of medical resources.

This study aims to develop five AI models that can assist in screening for positive metaphyseal signs on hand radiographs in a normal population. It may provide convenience and help us screen for KBD.

## Materials and methods

Patients

Medical records from individuals who had undergone right-hand X-ray screening for KBD were gathered from multiple KBD surveillance facilities in Shaanxi province. In accordance with the predefined inclusion criteria, 1,224 subjects were categorized into the metaphyseal positive group, while 10,126 subjects were included in the control group. The inclusion criteria were as follows: (1) age between 7 and 12 years old; (2) clear and complete right-hand X-ray film data; (3) no osteoarthritis, rheumatoid arthritis, gout, rickets, cretinism, primary dwarfism, metaphyseal dysplasia, achondroplasia, or other diseases; (4) individuals exhibiting diagnostic metaphyseal features of KBD were categorized into the metaphyseal positive group, whereas others were assigned to the control group. A positive metaphyseal sign is characterized by the presence of depressions in the metaphyseal calcification area on hand X-rays, which may also be accompanied by displacement of the epiphyseal nucleus or narrowing of the epiphyseal line and could include structural disruptions such as alterations in bone trabeculae [9].

Image preparation

All imaging data of the metaphyseal sign positive group and control group were obtained from Shaanxi province KBD screening. All radiographs were scanned (Microtek, Shanghai, China) and saved in high-pixel portable network graphics (PNG) format. All images were preprocessed. The images of the metaphyseal sign positive group were data-enhanced by 10 times. Firstly, each image in this group was resized to 360 × 360 pixels and randomly cropped to 256 × 256 pixels. Secondly, each image was randomly flipped left and right, and up and down. Thirdly, each image was randomized for brightness and contrast. Finally, we collected 12,240 preprocessed metaphyseal sign positive images. The control group was preprocessed similarly, and 10,126 control images were collected. Thus, we obtained a KBD metaphyseal sign diagnostic dataset (22,366 images) with 12,240 metaphyseal sign positive images and 10,126 control images.

Development of five metaphyseal sign diagnostic models for KBD

**Figure 1:**
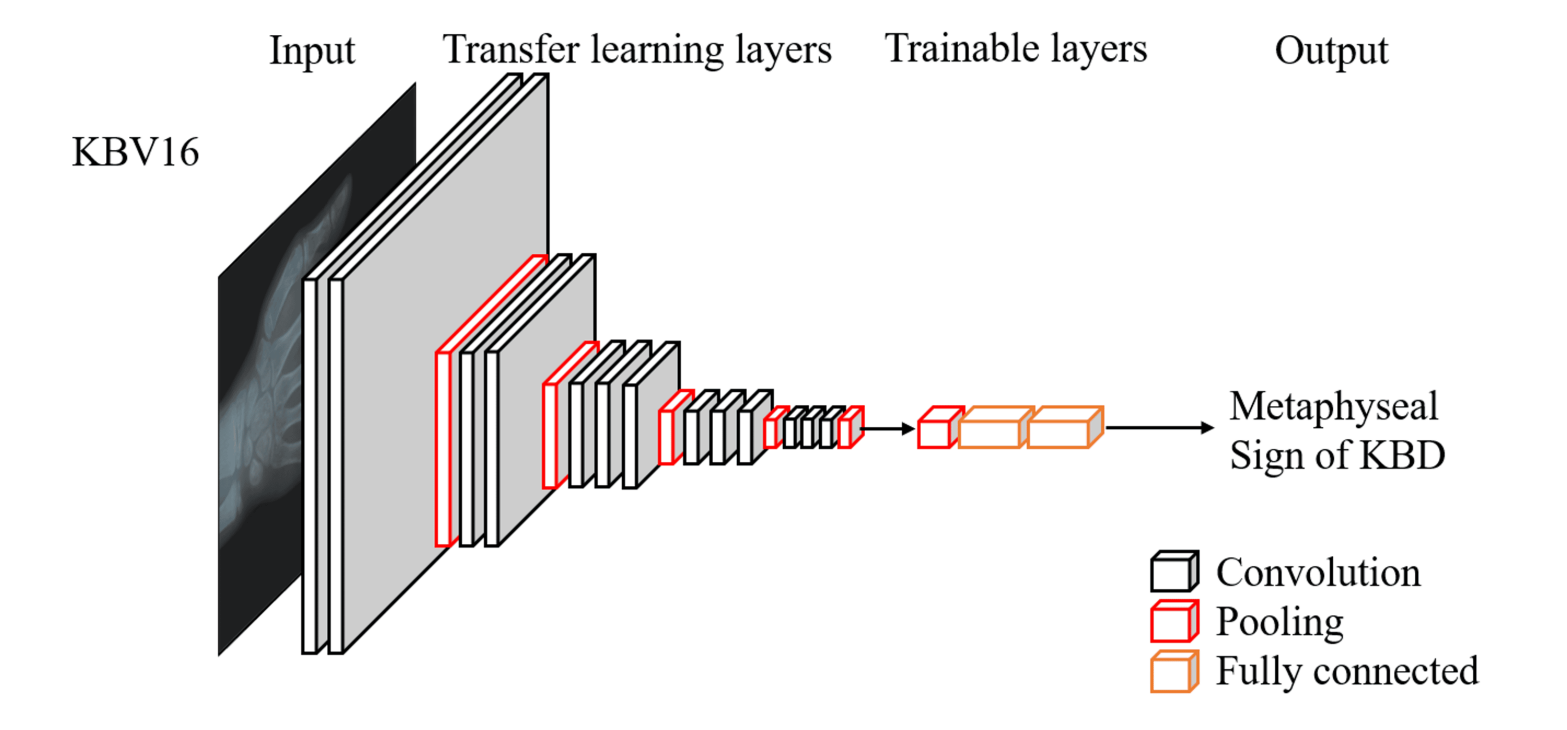
Architecture of KBV16 Image credit: Dr. Chao Li KBD: Kashin-Beck disease

Five classical AI pre-trained models, including VGG16, Xception, VGG19, Inception, and MobileNetV2, were employed in this study [14-17]. Based on the parameters of these pre-trained models, and after modification, we deployed five transfer learning models: KBV16 (Figure 1), KBX, KBV19, KBIn, and KBM2, which could diagnose KBD metaphyseal signs on hand X-rays. When learning stalled, we used the callback function during training to slow down the learning rate (LR).

KBV16 is based on the VGG16 architecture, which is known for its deep convolutional layers and ability to capture complex features from images. This model is particularly effective in extracting detailed texture and shape information from X-ray images. The VGG16 architecture is well-suited for identifying the subtle changes in bone structure and metaphyseal signs characteristic of KBD. Its deep layers can detect irregularities in bone margins, sclerosis, and the cone-shaped, fused, or fragmented metaphysis that are diagnostic of KBD.

KBX is based on the Xception model, which is designed to efficiently process high-resolution images and is particularly effective in capturing spatial hierarchies and contextual information. Xception's ability to capture spatial hierarchies makes it suitable for identifying the localized tissue necrosis and disturbed mineralization seen in KBD. It can effectively differentiate between normal and diseased bone structures.

KBV19 is based on the VGG19 model. Similar to VGG16, VGG19 has an even deeper architecture, which allows it to capture more complex features from the images. However, its deeper layers can also lead to increased computational complexity and potential overfitting if not properly regularized. VGG19's deeper layers can capture more detailed features of bone lesions, including the characteristic changes in the metaphysis and epiphysis associated with KBD.

KBIn is based on the Inception model. The Inception model is designed to capture features at multiple scales, making it effective in identifying both fine and coarse details in images. Inception's multi-scale feature extraction is beneficial for identifying the varied manifestations of KBD, including irregularities in bone margins and the presence of bone lesions at different scales.

KBM2 is based on the MobileNetV2 model. MobileNetV2 is designed for efficient computation and is particularly useful for applications where computational resources are limited. It uses depthwise separable convolutions to reduce the number of parameters and computations. Despite its efficiency, MobileNetV2 can still capture the essential features of bone lesions and KBD, making it a viable option for resource-constrained environments.

Comparison of five metaphyseal sign diagnostic models for KBD

At a ratio of 2:1:1, the dataset was randomly divided into a training set (11,182 images), validation set (5,592 images), and test set (5,592 images). Then, we trained five metaphyseal sign diagnostic models for 50 epochs individually and compared their performance under different LRs and batch size (BS) on the validation set. Finally, on the test set, we evaluated the performance of the optimized metaphyseal sign diagnostic model.

Model optimization

To develop and compare the performance of the deep learning models for diagnosing KBD using pediatric hand radiographs, we focused on optimizing key hyperparameters, including the LR and BS. These hyperparameters play a critical role in the training process and significantly impact the models' performance.

The LR determines the step size at each iteration while moving toward a minimum of a loss function. A higher LR can lead to faster convergence but may also overshoot the optimal solution, while a lower LR can result in more precise convergence but at the cost of increased training time. We experimented with a range of LRs to identify the optimal value for each model. The LRs tested included 0.001, 0.002, 0.004, 0.008, and 0.016. The selection of these values was based on common practices in LRs and preliminary experiments to ensure a wide range of possibilities. We monitored the training and validation loss for each LR to determine the optimal value. Specifically, we looked for an LR that provided a good balance between convergence speed and stability. The LR that resulted in the lowest validation loss and highest accuracy was selected for each model.

The BS affects the gradient estimation and the update frequency of the model parameters. A larger BS generally provides a more accurate estimate of the gradient but requires more memory and can lead to overfitting. Conversely, a smaller BS introduces more noise in the gradient estimation but can help in generalizing better. We tested different BS values to evaluate their impact on model performance. The BS values tested included 4, 8, 16, and 32. These values were chosen based on standard practices and the computational resources available. We compared the training and validation accuracy for each BS value. The BS value that achieved the highest validation accuracy while maintaining reasonable training time was selected. We also considered the trade-off between computational efficiency and model performance.

Statistical analysis

Two parameters, Binary Cross-Entropy (BCE) and Accuracy (ACC), were used to train models and evaluate their performance, respectively. The BCE loss function, as a loss function in this study, is a function that measures the difference between the probability distribution predicted by the model and the probability distribution of true labels. ACC, by calculating the ratio of the number of samples correctly predicted by the model to the total number of samples in the entire dataset, provides an intuitive way to measure the performance of the model. The closer these two values are to one, the better the model performs. The training and evaluation were conducted using a high-performance computing environment with NVIDIA Graphics Processing Units (GPUs) (NVIDIA, Santa Clara, CA, USA). All data operations were performed on TensorFlow 2.0.0 (Google, Inc., Mountain View, CA, USA).

## Results

Optimization of the KBV16 model

The performance of KBV16 under different LR and BS on the validation set is shown in Table 1, and we found that when the LR was 0.001 and the BS was 16, the KBV16 model achieved the best ACC of 0.9563 on the validation set.

**Table 1:** Performance (ACC) of KBV16 model under different LR and BS ACC: Accuracy; LR: Learning Rate; BS: Batch Size

BS	LR				
	0.001	0.002	0.004	0.008	0.016
4	0.9499	0.9433	0.9416	0.9485	0.9325
8	0.9478	0.9420	0.9523	0.9367	0.9299
16	0.9563	0.9505	0.9497	0.9501	0.9404
32	0.9501	0.9365	0.9470	0.9480	0.9459

**Figure 2:**
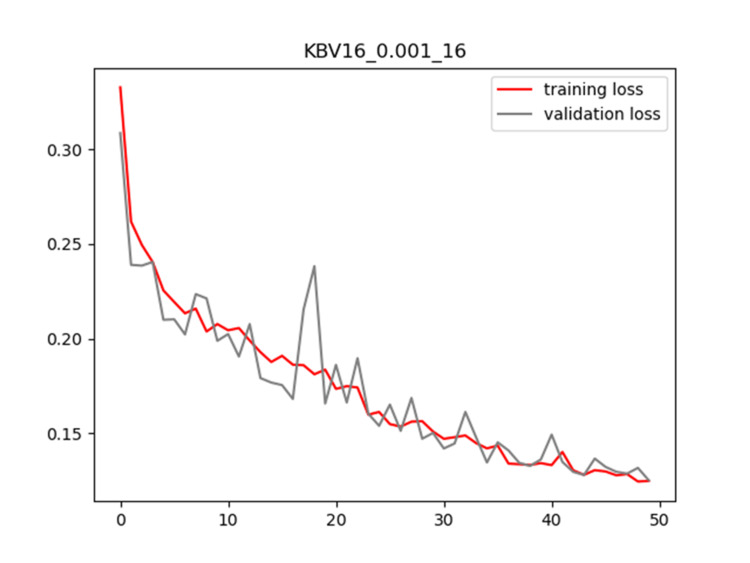
Plot of training and validation loss value for LR = 0.001 and BS = 16 for KBV16 model LR: Learning Rate; BS: Batch Size

**Figure 3:**
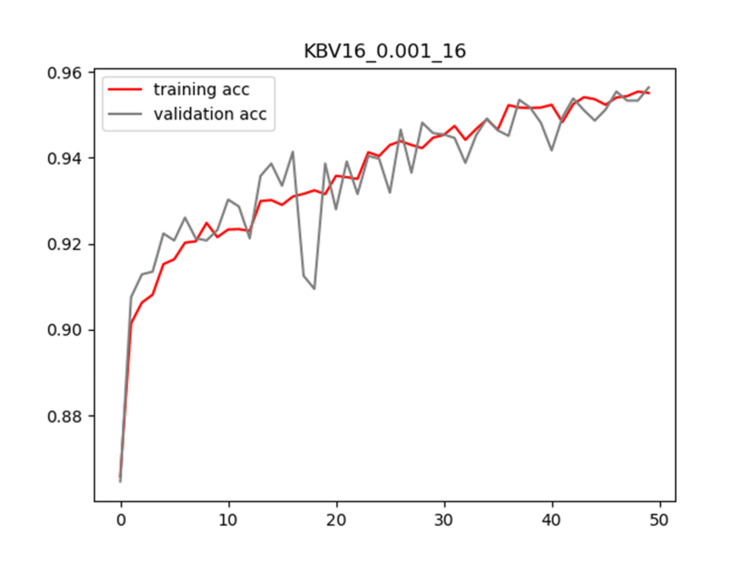
Plot of training and validation ACC for LR = 0.001 and BS = 16 for KBV16 model ACC: Accuracy; LR: Learning Rate; BS: Batch Size

Plots of training and validation loss and ACC for LR = 0.001 and BS = 16 for KBV16 are shown in Figures 2-3, respectively.

Optimization of the KBX model

The performance of KBX under different LR and BS on the validation set is shown in Table 2, and we found that when the LR was 0.002 and the BS was 16, the KBX model achieved the best ACC of 0.9054 on the validation set.

**Table 2:** Performance (ACC) of KBX model under different LR and BS ACC: Accuracy; LR: Learning Rate; BS: Batch Size

BS	LR				
	0.001	0.002	0.004	0.008	0.016
4	0.8929	0.8751	0.8760	0.8872	0.8905
8	0.8926	0.8843	0.8890	0.8908	0.8950
16	0.9017	0.9054	0.9051	0.8840	0.8996
32	0.8955	0.8903	0.8959	0.8840	0.8745

**Figure 4:**
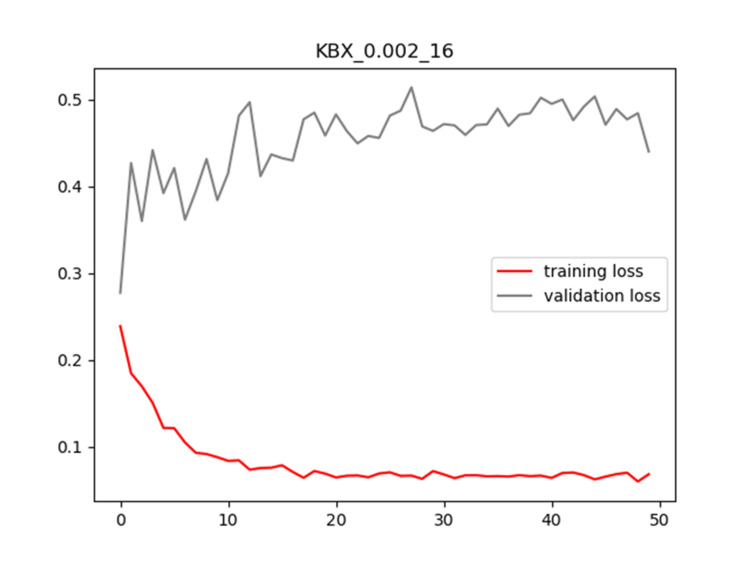
Plot of training and validation loss value for LR = 0.002 and BS = 16 for KBX model LR: Learning Rate; BS: Batch Size

**Figure 5:**
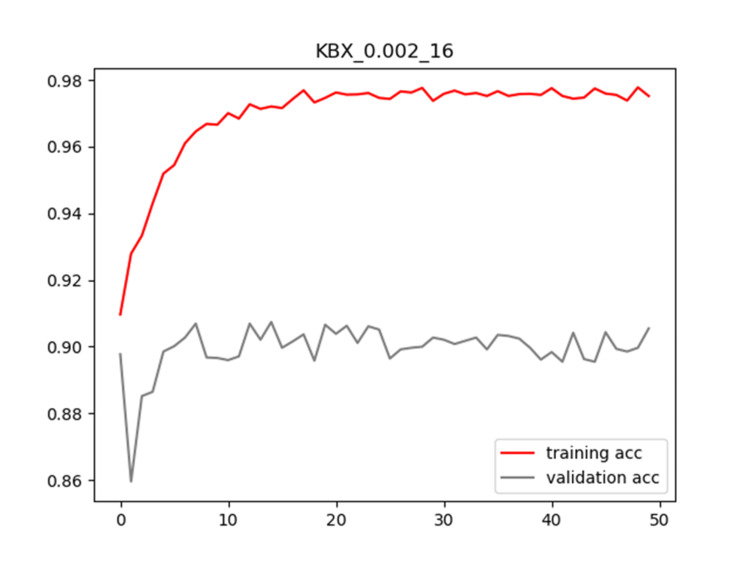
Plot of training and validation ACC for LR = 0.002 and BS = 16 for KBX model ACC: Accuracy; LR: Learning Rate; BS: Batch Size

Plots of training and validation loss and ACC for LR = 0.002 and BS = 16 for KBX are shown in Figures 4-5, respectively.

Optimization of the KBV19 model

The performance of KBV19 under different LR and BS on the validation set is shown in Table 3, and we found that when the LR was 0.002 and the BS was 32, the KBV19 model achieved the best ACC of 0.9343 on the validation set.

**Table 3:** Performance (ACC) of KBV19 model under different LR and BS ACC: Accuracy; LR: Learning Rate; BS: Batch Size

BS	LR				
	0.001	0.002	0.004	0.008	0.016
4	0.9292	0.9316	0.9261	0.9269	0.9272
8	0.9282	0.9261	0.9299	0.9325	0.9254
16	0.9278	0.9320	0.9306	0.9225	0.9243
32	0.9327	0.9343	0.9304	0.9280	0.9262

**Figure 6:**
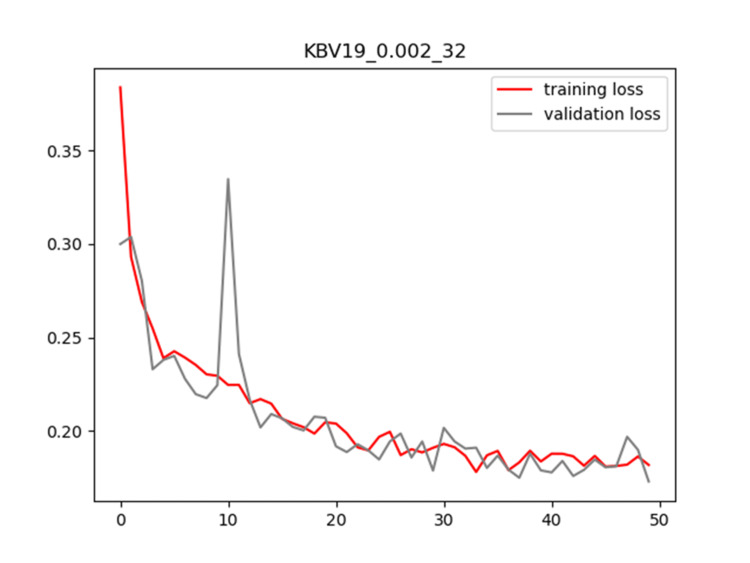
Plot of training and validation loss value for LR = 0.002 and BS = 32 for KBV19 model LR: Learning Rate; BS: Batch Size

**Figure 7:**
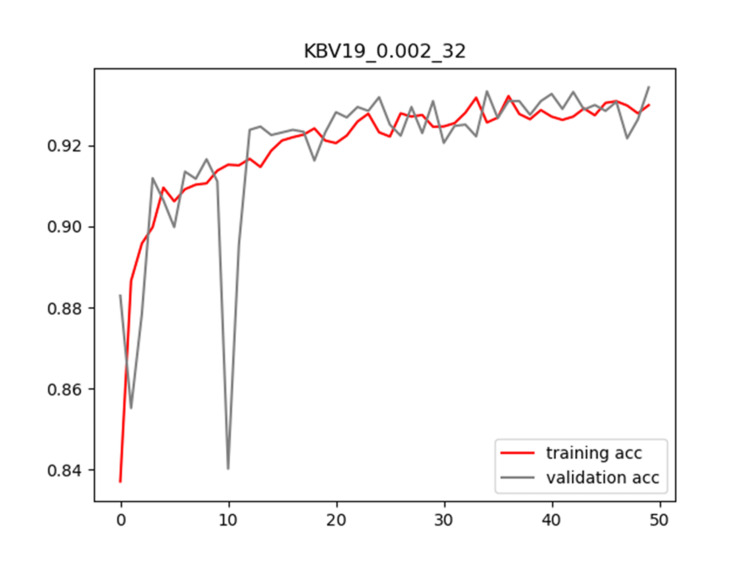
Plot of training and validation ACC for LR = 0.002 and BS = 32 for KBV19 model ACC: Accuracy; LR: Learning Rate; BS: Batch Size

Plots of training and validation loss and ACC for LR = 0.002 and BS = 32 for KBV19 are shown in Figures 6-7, respectively.

Optimization of the KBIn model

The performance of KBIn under different LR and BS on the validation set is shown in Table 4, and we found that when the LR was 0.016 and the BS was 4, the KBIn model achieved the best ACC of 0.8669 on the validation set. Table 4 illustrates the performance of the KBIn model across various LR and BS on the validation dataset. It was observed that the optimal ACC of 0.8669 for the KBIn model on the validation set was achieved when the LR was set to 0.016 and the BS was 4.

**Table 4:** Performance (ACC) of KBIn model under different LR and BS ACC: Accuracy; LR: Learning Rate; BS: Batch Size

BS	LR				
	0.001	0.002	0.004	0.008	0.016
4	0.8197	0.8434	0.8622	0.8577	0.8669
8	0.7885	0.7582	0.8526	0.8186	0.8571
16	0.7434	0.7473	0.7283	0.7320	0.8061
32	0.6889	0.6947	0.7366	0.7126	0.7700

**Figure 8:**
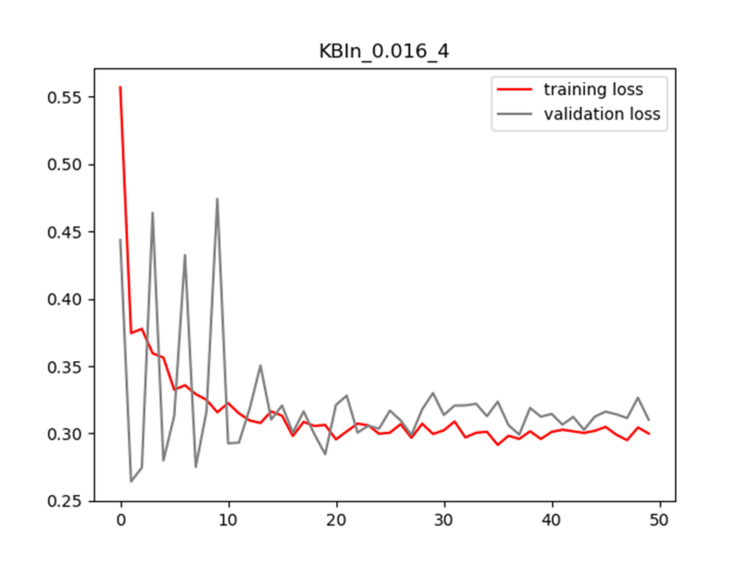
Plot of training and validation loss value for LR = 0.016 and BS = 4 for KBIn model LR: Learning Rate; BS: Batch Size

**Figure 9:**
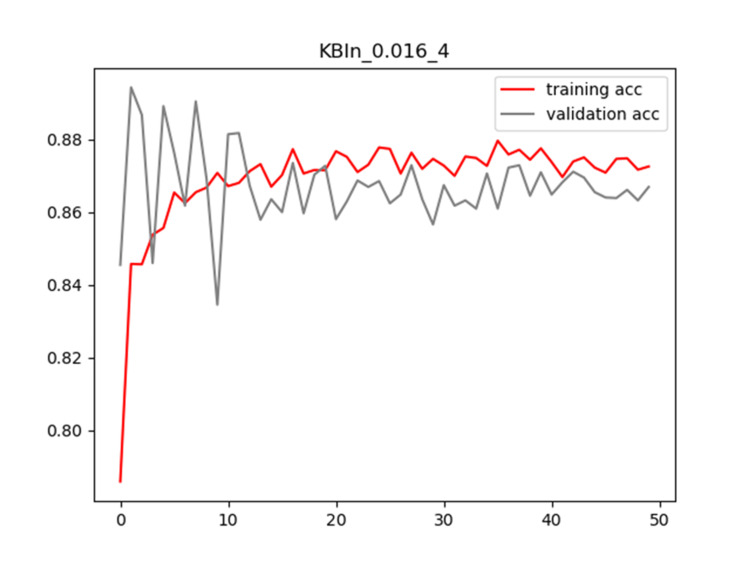
Plot of training and validation ACC for LR = 0.016 and BS = 4 for KBIn model ACC: Accuracy; LR: Learning Rate; BS: Batch Size

Plots of training and validation loss and ACC for LR = 0.016 and BS = 4 for KBIn are shown in Figures 8-9, respectively.

Optimization of the KBM2 model

As presented in Table 5, the KBM2 model's performance was evaluated under diverse LR and BS within the validation dataset. The optimal accuracy of 0.8284 for the KBM2 model on the validation set was achieved when the LR was set to 0.001 and the BS was 4.

**Table 5:** Performance (ACC) of KBM2 model under different LR and BS ACC: Accuracy; LR: Learning Rate; BS: Batch Size

BS	LR				
	0.001	0.002	0.004	0.008	0.016
4	0.8284	0.7790	0.7878	0.7434	0.7489
8	0.7858	0.7506	0.7990	0.7666	0.8188
16	0.7946	0.7941	0.7603	0.7637	0.7487
32	0.6967	0.8094	0.7107	0.7316	0.7345

**Figure 10:**
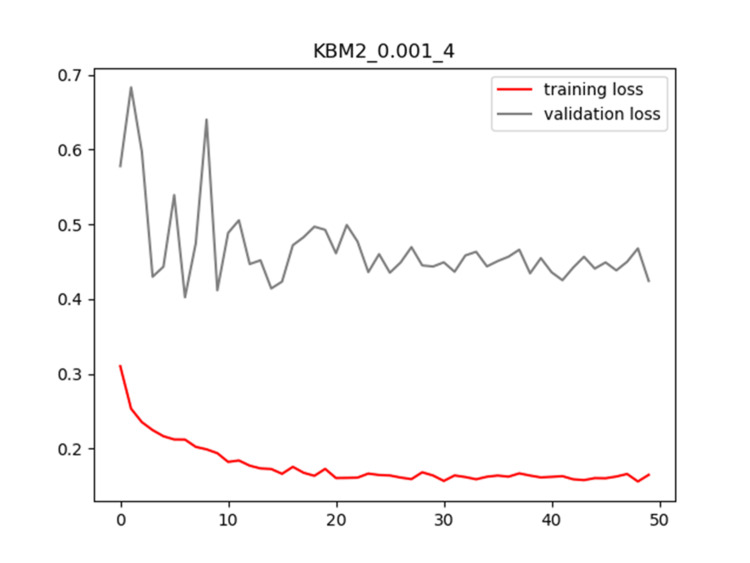
Plot of training and validation loss value for LR = 0.001 and BS = 4 for KBM2 model LR: Learning Rate; BS: Batch Size

**Figure 11:**
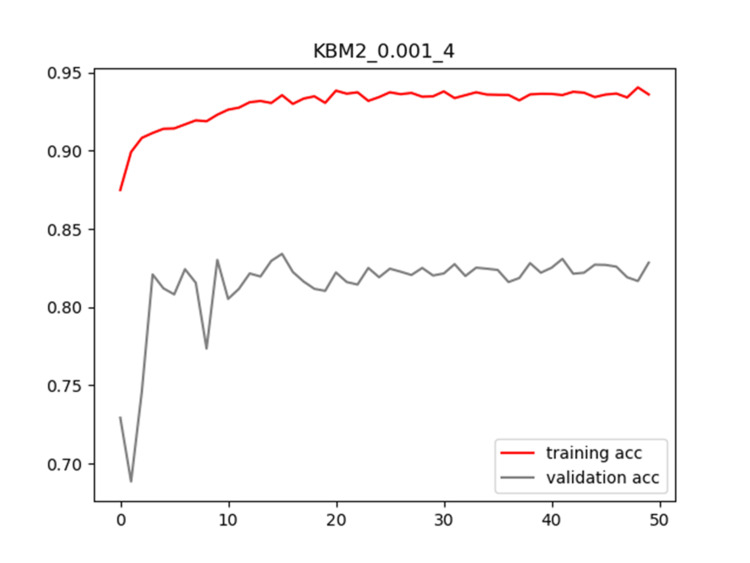
Plot of training and validation ACC for LR = 0.001 and BS = 4 for KBM2 model ACC: Accuracy; LR: Learning Rate; BS: Batch Size

Plots of training and validation loss and ACC for LR = 0.001 and BS = 4 for KBM2 are shown in Figures 10-11, respectively.

Comparison of five optimized models and their final performance on test set

After evaluating the performance of the five refined models under different LR and BS on the validation dataset, it was observed that the KBV16 model outperformed the others. Finally, the optimized KBV16 model achieved an ACC of 0.9535 on the test set.

## Discussion

In this research, we assessed the performance of five deep learning models, namely KBV16, KBX, KBV19, KBIn, and KBM2, to assist in identifying metaphyseal signs associated with KBD in pediatric hand radiographs. Utilizing transfer learning techniques, we optimized these models and assessed their performance on a test set. The KBV16 model demonstrated superior performance, achieving an accuracy of 0.9563 on the validation set and 0.9535 on the test set, showcasing its high stability and broad generalizability [18]. These results indicated that the KBV16 model was capable of effectively identifying characteristics of KBD from X-ray images, serving as an effective and beneficial tool for clinical diagnosis.

As a key characteristic for diagnosing KBD, metaphyseal signs are essential for training models to interpret X-ray images [19]. In our study, we utilized data augmentation strategies, including random cropping and flipping, which improved the model's capability to adapt to X-rays with diverse angles and positions, thereby enhancing the model's feature generalization [20]. Moreover, our findings indicated that employing a lower LR and a moderate BS led to improved model performance on the validation set [21]. These insights highlight the importance of feature selection and data augmentation techniques in enhancing model performance.

Despite the promising performance of the KBV16 model, further considerations are still needed regarding its computational efficiency and real-time performance, especially for implementation in medical environments with limited resources [22]. Future research could benefit from exploring the combination of deep learning with expert systems to enhance diagnostic precision and interpretability [23]. Given the complexity of KBD, it is also essential for future research to develop predictive models for disease staging and personalized treatment strategies [24].

There are a large number of patients with rare diseases worldwide, and about half of the suspected cases remain undiagnosed, with a long diagnostic cycle [25]. AI technology has shown great potential in the diagnosis of rare diseases [26]. By analyzing patients' clinical phenotypes and genetic data, it can quickly screen for potential pathogenic variants. AI technology has made significant progress in the diagnosis of rare diseases [27]. These studies provide rich experience and technical support for the application of AI in the diagnosis of rare diseases. The application of AI technology in the diagnosis of KBD can not only improve diagnostic efficiency and accuracy, but also optimize the allocation of medical resources and reduce human effort. Moreover, the successful application of AI in the diagnosis of rare diseases provides important references and experiences for our study. Future research should further optimize AI models and integrate them better into existing healthcare systems to improve the quality and accessibility of medical services.

The findings of this study present new possibilities for the early diagnosis and treatment of KBD. Utilizing deep learning models, the rapid and accurate identification of KBD characteristics from X-rays becomes feasible, which is essential for increasing diagnostic rates and reducing misdiagnoses. With ongoing advancements in technology, these models could be integrated into mobile devices, facilitating rapid on-site diagnosis and improving medical services for patients in remote locations.

Although our research provided some valuable findings, several limitations must be recognized. Firstly, the sample size and demographic scope of our study may limit the broader applicability of our findings. To strengthen external validity, future research should aim for larger and more diverse samples that can better represent various populations. Secondly, the lack of longitudinal data in our research restricts our capacity to monitor the long-term progression of KBD and assess the sustained effects of treatments. Studies with longer follow-up periods are necessary for a more profound understanding of the disease’s natural history. Thirdly, manual interpretation of X-rays may introduce subjectivity and variability, potentially affecting the consistency and reliability of metaphyseal change assessments. Integrating advanced imaging and AI technologies could improve the precision and uniformity of diagnostic procedures. Lastly, our study did not differentiate between various stages of KBD, which may have implications for the specificity of our diagnostic and prognostic insights. Future models that incorporate disease staging could offer more refined diagnostic capabilities and prognostic predictions.

## Conclusions

In summary, this study developed five deep-learning models that have proven to be effective in assisting the diagnosis of KBD metaphyseal signs on pediatric hand radiographs. The KBV16 model, distinguished by its high accuracy and stability, emerges as an influential tool for the early detection of KBD. Future research should focus on further optimizing these models and exploring their practical applications in clinical settings. Additionally, we should pay attention to the model’s computational efficiency and real-time capabilities, as well as to strategies for integrating these technologies with current healthcare systems to provide better medical services to patients.

## References

[1] Zhang L (2021). Treatment and research progress in Kashin-Beck disease. Chin J Endemiol.

[2] Zhang G, Liang C, Ma Y (2022). Step treatment of Kashin-Beck disease arthritis of the knee. Chin J Endemiol.

[3] Wang K, Yu J, Liu H (2020). Endemic Kashin-Beck disease: a food-sourced osteoarthropathy. Semin Arthritis Rheum.

[4] Cui SL, Liu H, Pei JR (2024). Summary analysis of national surveillance on Kashin-Beck disease from 1990 to 2023. Biomed Environ Sci.

[5] Wang Z, Chen Q (2016). Research progress made in Kashin-Beck disease. Chin J Endemiol.

[6] Xianhao W, Xin Z, Yun C (2021). Whether the limitation of the phalangeal epiphysis "before the equal-diameter period" should be extended to "before the ultra-diameter period" in X-ray diagnosis of Kashin-Beck disease. Chin J Endemiol.

[7] Xiong G (2001). Diagnostic, clinical and radiological characteristics of Kashin-Beck disease in Shaanxi Province, PR China. Int Orthop.

[8] Zeng Y, Zhou Z, Shen B (2014). X-ray image characteristics and related measurements in the ankles of 118 adult patients with Kashin-Beck disease. Chin Med J (Engl).

[9] National Health Commission of the People's Republic of China (2010). Diagnosis of Kaschin-Beck Disease (WS_T 207-2010).

[10] Chen X, Liu H, Wen M (2023). Epidemiological investigation of Kaschin-Beck disease prevention and control in Fu County, Shaanxi Province from 1954 to 2022. Chin J Endemiol.

[11] National Health Commission of the People's Republic of China (2010). 9. NHCC: Criteria for delimitation and classification of Kashin-Beck disease endemic area: GB 16395-2011. Criteria for Delimitation and Classification of Kashin-Beck Disease Endemic Area (GB 16395-2011).

[12] Cui S, Pei J, Jiao Z (2023). Summary report of a national survey of Kashin-Beck disease prevalence in 2020. Chin J Endemiol.

[13] Fei X, Chen X, Wang Y (2022). Epidemic trend of Kaschin-Beck disease in Gansu Province from 2004 to 2018. Chin J Endemiol.

[14] Omiotek Z, Kotyra A (2021). Flame image processing and classification using a pre-trained VGG16 model in combustion diagnosis. Sensors (Basel).

[15] Rahimzadeh M, Attar A (2020). A modified deep convolutional neural network for detecting COVID-19 and pneumonia from chest X-ray images based on the concatenation of Xception and ResNet50V2. Inform Med Unlocked.

[16] Han B, Du J, Jia Y, Zhu H (2021). Zero-watermarking algorithm for medical image based on VGG19 deep convolution neural network. J Healthc Eng.

[17] Ekmekyapar T, Taşcı B (2023). Exemplar MobileNetV2-based artificial intelligence for robust and accurate diagnosis of multiple sclerosis. Diagnostics (Basel).

[18] Kim HE, Cosa-Linan A, Santhanam N, Jannesari M, Maros ME, Ganslandt T (2022). Transfer learning for medical image classification: a literature review. BMC Med Imaging.

[19] Suganya A, Aarthy SL (2023). Application of deep learning in the diagnosis of Alzheimer’s and Parkinson’s disease: a review. Curr Med Imaging.

[20] Shorten C, Khoshgoftaar TM (2019). A survey on image data augmentation for deep learning. J Big Data.

[21] He K, Zhang X, Ren S, Sun J (2016). Deep residual learning for image recognition. 2016 IEEE Conference on Computer Vision and Pattern Recognition (CVPR).

[22] Esteva A, Kuprel B, Novoa RA, Ko J, Swetter SM, Blau HM, Thrun S (2017). Dermatologist-level classification of skin cancer with deep neural networks. Nature.

[23] Rajkomar A, Oren E, Chen K (2018). Scalable and accurate deep learning with electronic health records. NPJ Digit Med.

[24] Miotto R, Li L, Kidd BA, Dudley JT (2016). Deep patient: an unsupervised representation to predict the future of patients from the electronic health records. Sci Rep.

[25] Haendel M, Vasilevsky N, Unni D (2020). How many rare diseases are there?. Nat Rev Drug Discov.

[26] Brasil S, Pascoal C, Francisco R, Dos Reis Ferreira V, Videira PA, Valadão AG (2019). Artificial intelligence (AI) in rare diseases: is the future brighter?. Genes (Basel).

[27] Wojtara M, Rana E, Rahman T, Khanna P, Singh H (2023). Artificial intelligence in rare disease diagnosis and treatment. Clin Transl Sci.

